# Supplementary material for: LIN-23 Affects C. elegans Pathogen and Stress Resistance by Modulating SKN-1 Activity
Source: bioRxiv. 2026 Jul 26:2026.07.22.740106. Preprint. [Version 1] doi: 10.64898/2026.07.22.740106 (PMC13420414; doi:10.64898/2026.07.22.740106)
Supplement: 1 [file NIHPP2026.07.22.740106v1-supplement-1.pdf]

bioRxiv preprint doi: https://doi.org/10.64898/2026.07.22.740106; this version posted July 26, 2026. The copyright holder for this preprint (which was not certified by peer review) is the author/funder, who has granted bioRxiv a license to display the preprint in perpetuity. It is made available under a CC-BY 4.0 International license.

Supplemental Fig.1 Effects of LIN-23 on SKN-1 nuclear localization during *P. aeruginosa* exposure. a) Wild-type and *lin-23(ot1)* worms containing an integrated SKN-1B/C::GFP transgene were exposed to *P. aeruginosa* or *E. coli*, and SKN-1B/C::GFP localization was examined by fluorescence microscopy. Representative intestinal nuclei exhibiting nuclear localization of SKN-1B/C::GFP are indicated by white arrows. Scale bars represent 50 µm. b) SKN-1C nuclear localization was scored based on the GFP signal and categorized as low, medium, or high. The percentage for each category was quantified, and the number of worms used in scoring each experimental condition is indicated (*n*). Levels of intestinal SKN-1B/C::GFP nuclear localization in *lin-23(ot1)* mutants and wild-type animals treated with *lin-23* or *skn-1* RNAi were compared to their respective controls. ****P < 0.0001; ns, not significant.

Supplemental Fig. 2 WDR-23B::wrmScarlet expressed under endogenous promoter. a) Representative images of WDR-23B::wrmScarlet expressed under the endogenous *wdr-23* promoter in wild-type background following exposure to *E. coli* or *E. faecalis*. Representative intestinal nuclei are outlined with white dashed lines. WDR-23B

bioRxiv preprint doi: https://doi.org/10.64898/2026.07.22.740106; this version posted July 26, 2026. The copyright holder for this preprint (which was not certified by peer review) is the author/funder, who has granted bioRxiv a license to display the preprint in perpetuity. It is made available under a CC-BY 4.0 International license.

fluorescence was not detected under the endogenous promoter in either condition, likely due to low endogenous expression levels. b-c) Representative images of WDR-23B::wrmScarlet expressed under the intestine-specific *vha-6* promoter in wild-type and *lin-23(ot1)* backgrounds following exposure to *E. coli* or *E. faecalis*. These images are duplicates of the data presented in the main text and are included here to facilitate side-by-side comparison between genotypes.

bioRxiv preprint doi: https://doi.org/10.64898/2026.07.22.740106; this version posted July 26, 2026. The copyright holder for this preprint (which was not certified by peer review) is the author/funder, who has granted bioRxiv a license to display the preprint in perpetuity. It is made available under a CC-BY 4.0 International license.

Supplemental Figure 1

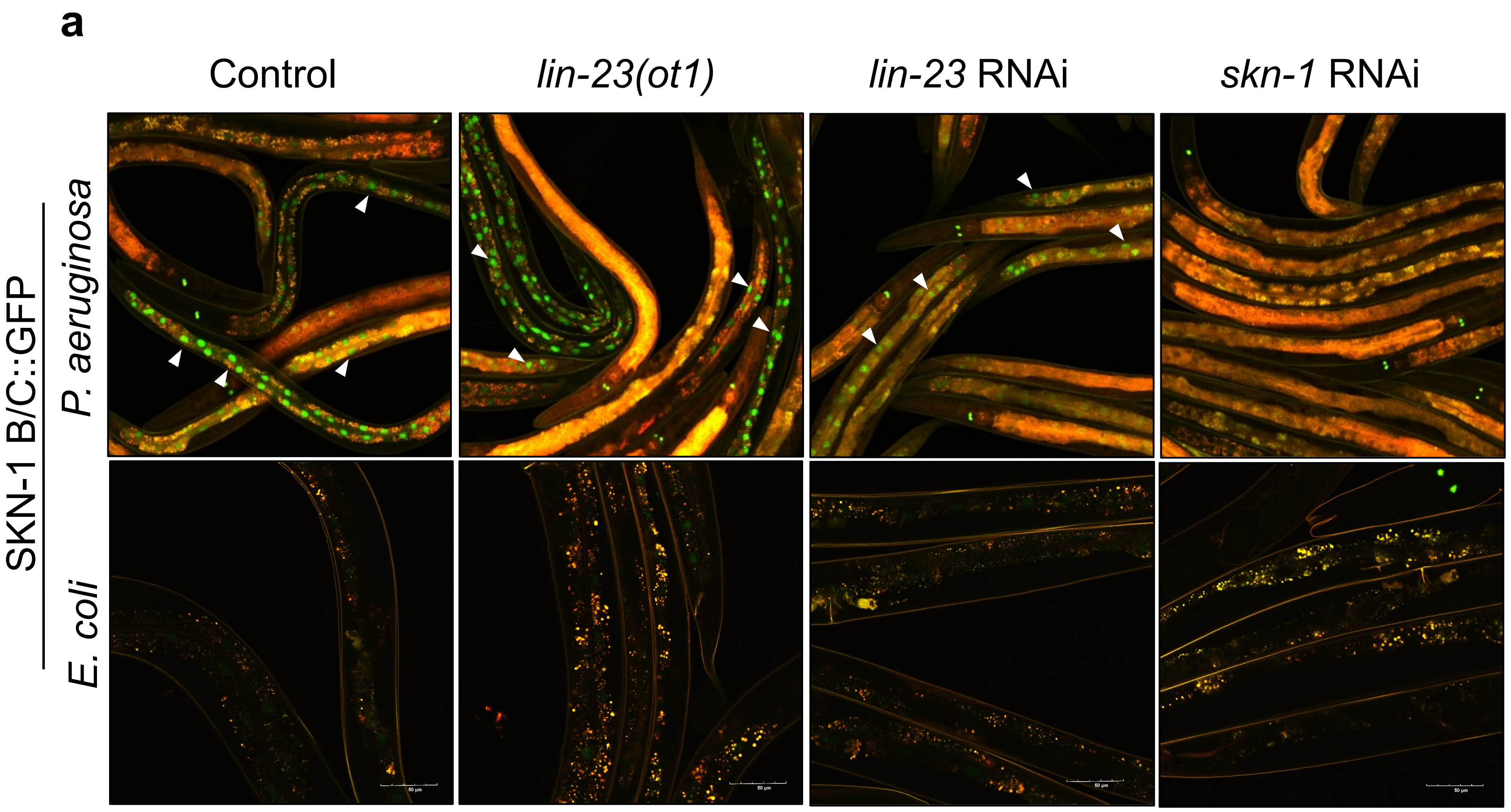

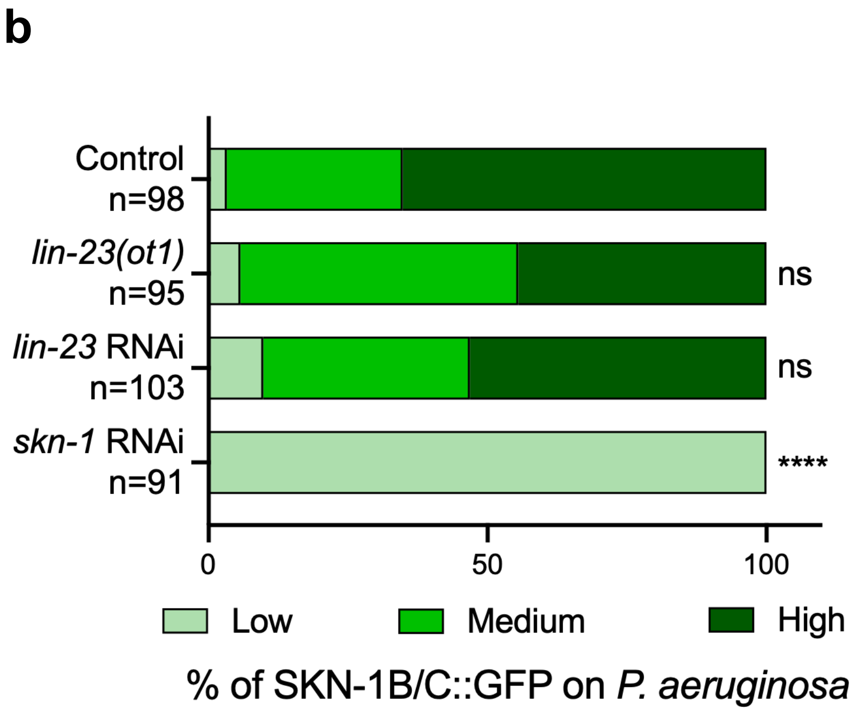

bioRxiv preprint doi: https://doi.org/10.64898/2026.07.22.740106; this version posted July 26, 2026. The copyright holder for this preprint (which was not certified by peer review) is the author/funder, who has granted bioRxiv a license to display the preprint in perpetuity. It is made available under a CC-BY 4.0 International license.

Supplemental Figure 2

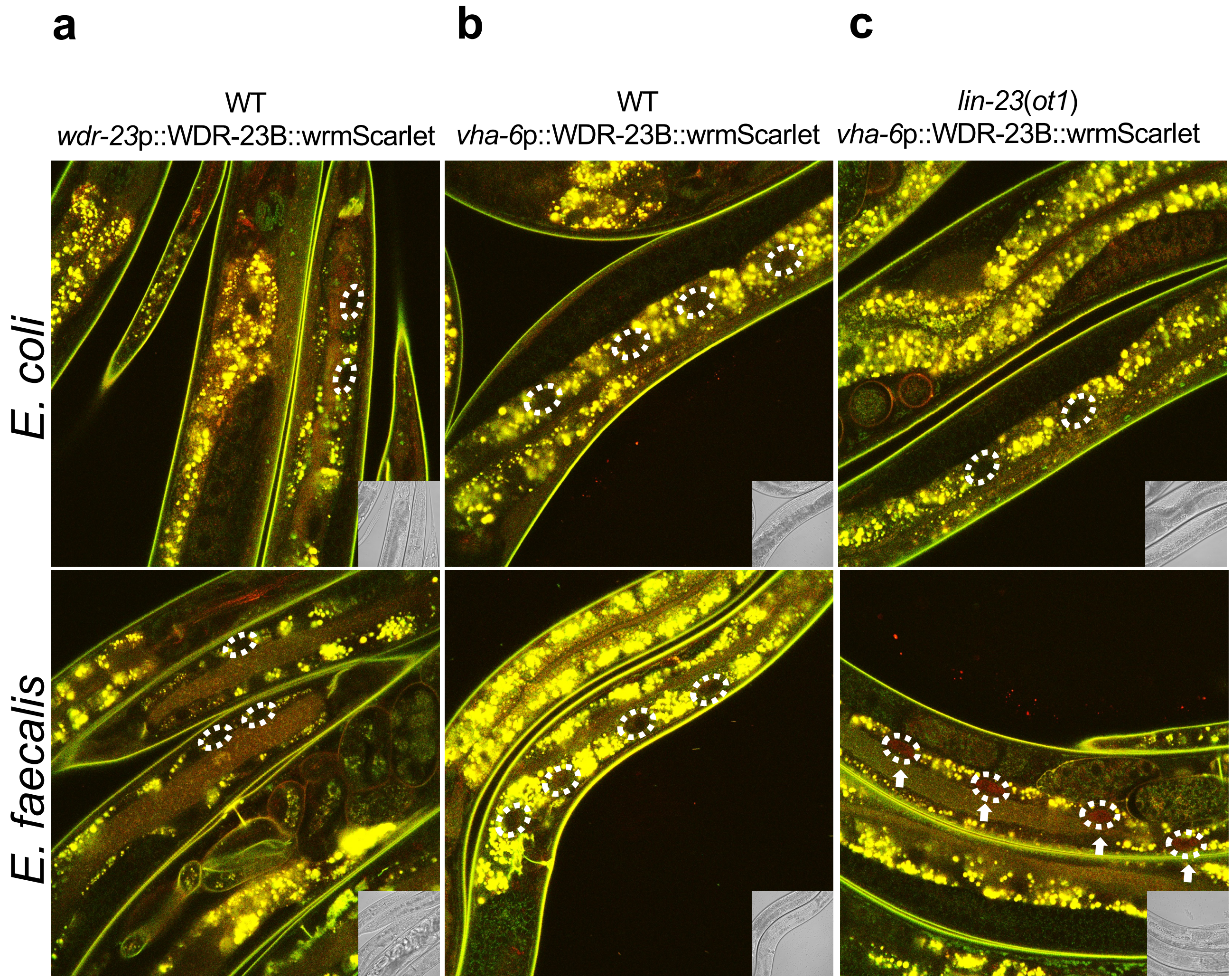

bioRxiv preprint doi: https://doi.org/10.64898/2026.07.22.740106; this version posted July 26, 2026. The copyright holder for this preprint (which was not certified by peer review) is the author/funder, who has granted bioRxiv a license to display the preprint in perpetuity. It is made available under aCC-BY 4.0 International license.

Supplemental Data: Western blot replicates

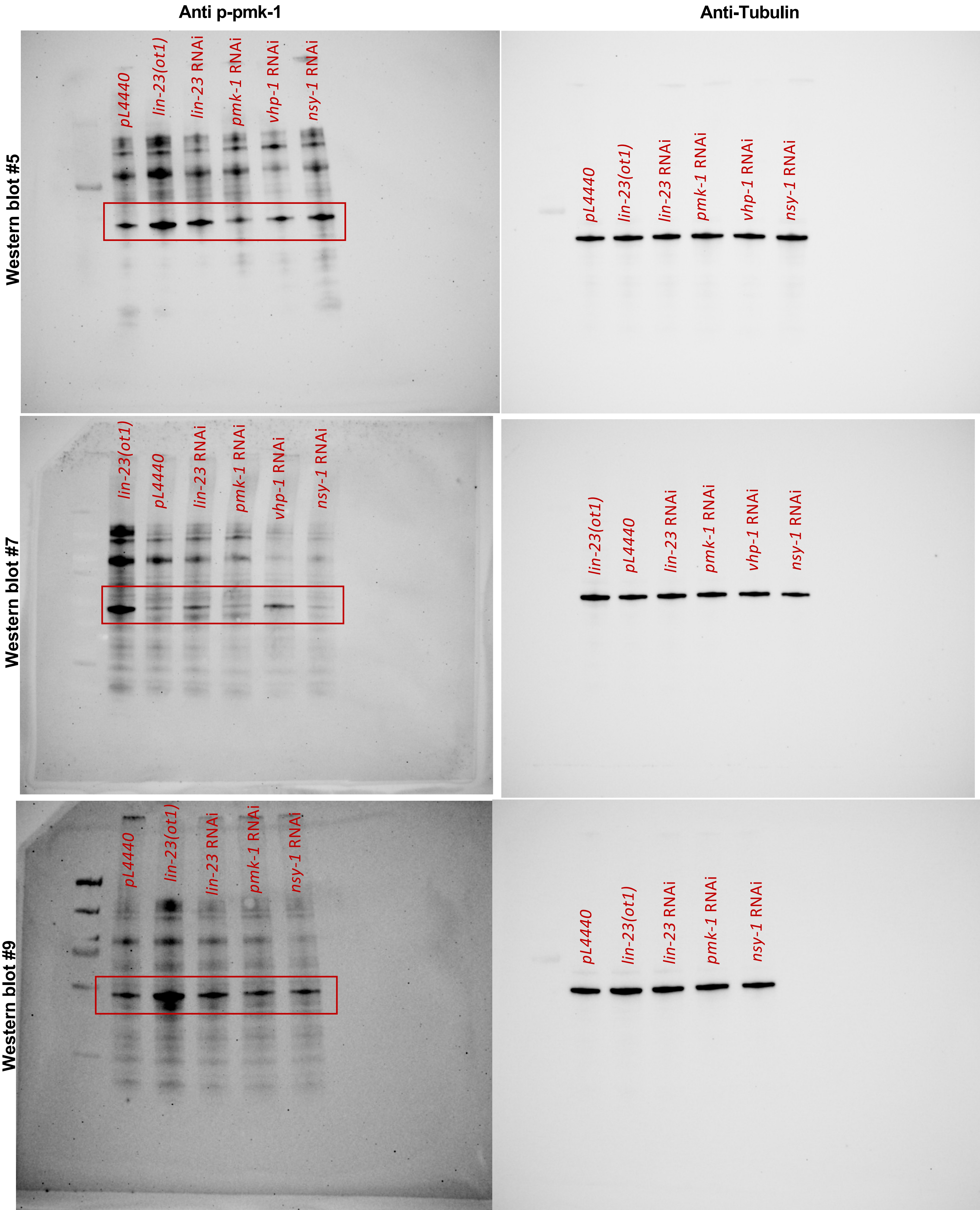

bioRxiv preprint doi: https://doi.org/10.64898/2026.07.22.740106; this version posted July 26, 2026. The copyright holder for this preprint (which was not certified by peer review) is the author/funder, who has granted bioRxiv a license to display the preprint in perpetuity. It is made available under a CC-BY 4.0 International license.

Supplementary Table 1		
**Strain**	**WormBase ID/Source**	**Description**
C. elegans		
N2	WBStrain00000001	Bristol, wild-type
OH1476	WBStrain00029321	*lin-23(ot1)* II; oxIs12 X
VP579	K. Choe PMCID: PMC2682033	*wdr-23(tm1817)*; dvIs19
CL2166	WBStrain00005102	*dvIs19 [(pAF15)gst-4p::GFP::NLS] III*
LD1171	WBStrain00024128	*ldIs3 [gcs-1p::gfp+pRF4(rol-6(su1006))]*
LD001	WBStrain00024124	*dIs7 [skn-1b/c::GFP + rol-6(su1006)]*
IG544	WBStrain00021982	*nipi-3(fr4) X*
COP2958	InVivo Biosystems, This study	[pNU3862 ([wdr-23p::WDR-23B::wrmScarlet::tbb-2u; rps-0p::HygR::unc-54u])) Chr. IV/(cxTi10882)))
COP2949	InVivo Biosystems, This study	[pNU3862 ([wdr-23p::WDR-23B::wrmScarlet::tbb-2u; rps-0p::HygR::unc-54u])) Chr. IV/(cxTi10882)))
GF250	This study	OH1476 backcrossed 4x to N2
GF251	This study	VP579 backcrossed 4x to N2
GF252	This study	*lin-23(ot1)* II; dvIs19 [(pAF15)gst-4p::GFP::NLS] III
GF253	This study	*lin-23(ot1)* II; ldIs3 [gcs-1p::gfp+pRF4(rol-6(su1006))]
GF254	This study	*lin-23(ot1)* II; dIs7 [skn-1b/c::GFP + rol-6(su1006)]
GF255	This study	*lin-23(ot1)* II; [pNU3863 ([vha-6p::WDR-23B::wrmScarlet::tbb-2u; rps-0p::HygR::unc-54u] Chr. IV/(cxti10882)
GF256	This study	*wdr-23(tm1817) I; lin-23(ot1) II*
Bacteria		
HT115(DE3)	WBStrain00041080	*E. coli*
OG1RF	WBStrain00041967	*E. faecalis*
OP50	WBStrain00041969	*E. coli*
PA14	WBStrain00041978	*P. aeruginosa*

bioRxiv preprint doi: https://doi.org/10.64898/2026.07.22.740106; this version posted July 26, 2026. The copyright holder for this preprint (which was not certified by peer review) is the author/funder, who has granted bioRxiv a license to display the preprint in perpetuity. It is made available under a CC-BY 4.0 International license.

Supplementary Table 2	
**qRT-PCR**	
Forward *act-1*	5’ - CCATCATGAAGTGCGACATTG
Reverse *act-1*	5’ - CATGGTTGATGGGGCAAGAG
Forward *gst-4*	5’ - AAAGCTGAAGCCAACGACTC
Reverse *gst-4*	5’ - TCTGCAGTTTTTCCAGCG
Forward *gcs-1*	5’ - AATGCCTTACGGAGGTCTC
Reverse *gcs-1*	5’ - AAGAGATGGGAACGATATCG
**Conventional PCR**	
Forward external *wdr-23(tm1817)*	5’ - CGTGGAACATCGATCTCAAAC
Reverse external *wdr-23(tm1817)*	5’ - CATCGATAGTCCCAGTGTTG
Forward internal *wdr-23(tm1817)*	5’ - CGGATAGCAGTGAACAAATCG
Reverse internal *wdr-23(tm1817)*	5’ - CGCAAAAGATACGGTACCTGC
Forward *lin-23(ot1)*	5’ - GTTCTTCATCTTCGATTTGC
Reverse *lin-23(ot1)*	5’ - GGATGCAAAAGGATACTG
Forward pNU3862 CEH8796	5’ - GAGGAAACTTTATTGTGCCGCC
Reverse pNU3862 CEH6995	5’ - CAATGTTTACTAGACCGGGGCT
**Sequencing**	
Forward *lin-23(ot1)*	5’ - TGCCGGGGACAATGATGAAT
Reverse *lin-23(ot1)*	5’ - GGTTGTGCGTGCGATATTCC

bioRxiv preprint doi: https://doi.org/10.64898/2026.07.22.740106; this version posted July 26, 2026. The copyright holder for this preprint (which was not certified by peer review) is the author/funder, who has granted bioRxiv a license to display the preprint in perpetuity. It is made available under a CC-BY 4.0 International license.

Figure 1C

Object mean GFP

gst-4 ::GFP representative graph valu	Control (E. coli)	Control	lin-23 RNAi	lin-23(ot1)	skn-1 RNAi
	14587.915	38254.11	32039.05333	26129.5	24517.495
	16676.33	35012.33333	27015.665	33468.5	33266
	20456.5	38876.44333	33934.665	29854	19826

PRISM ANALYSIS

Number of families	1
Number of comparisons per family	4
Alpha	0.05

Dunnett's multiple comparisons test	Mean diff.	95.00% CI of diff.	Below threshold?	Summary	Adjusted P Value	B-?	
Control vs. Control *(E. coli)*	20141	15337 to 24944	Yes	****	<0.0001	A	Control *(E. coli)*
Control vs. *lin-23* RNAi	6236	1643 to 10829	Yes	**	0.0053	C	*lin-23*
Control vs. lin-23(*ot1*)	7564	2760 to 12367	Yes	**	0.0012	D	lin-23(*ot1*)
Control vs. *skn-1* RNAi	11849	6372 to 17326	Yes	****	<0.0001	E	*skn-1*

Test details	Mean 1	Mean 2	Mean diff.	SE of diff.	n1	n2	q	DF
Control vs. Control *(E. coli)*	37381	17240	20141	1832	9	6	10.99	27
Control vs. *lin-23* RNAi	37381	31145	6236	1752	9	7	3.559	27
Control vs. lin-23(*ot1*)	37381	29817	7564	1832	9	6	4.128	27
Control vs. *skn-1* RNAi	37381	25532	11849	2089	9	4	5.672	27

bioRxiv preprint doi: https://doi.org/10.64898/2026.07.22.740106; this version posted July 26, 2026. The copyright holder for this preprint (which was not certified by peer review) is the author/funder, who has granted bioRxiv a license to display the preprint in perpetuity. It is made available under a CC-BY 4.0 International license.

Figure 1D

Object mean GFP

gcs-1 ::GFP representative graph val	Control (E. coli)	Control	lin-23 RNAi	lin-23(ot1)	skn-1 RNAi
	1025.666667	1881.333333	1368	1320.67	967.3333333
	639	1049.333333	669	790	690.6666667
	587.3333333	1252.666667	757	550	811

PRISM ANALYSIS

Number of families	1
Number of comparisons per family	4
Alpha	0.05

Dunnett's multiple comparisons test	Mean diff.	95.00% CI of diff.	Below threshold?	Summary	Adjusted P Value	B-?	
Control vs. Control $^{(E. coli)}$	643.8	273.1 to 1014	Yes	***	0.0003	A	Control $^{(E. coli)}$
Control vs. *lin-23* RNAi	463.1	92.41 to 833.8	Yes	*	0.0103	C	*lin-23*
Control vs. lin-23(*ot1*)	507.6	136.9 to 878.3	Yes	**	0.0045	D	lin-23(*ot1*)
Control vs. *skn-1* RNAi	571.4	200.7 to 942.1	Yes	**	0.0013	E	*skn-1*

Test details	Mean 1	Mean 2	Mean diff.	SE of diff.	n1	n2	q	DF
Control vs. Control $^{(E. coli)}$	1394	750.7	643.8	145.8	9	9	4.416	40
Control vs. *lin-23* RNAi	1394	931.3	463.1	145.8	9	9	3.177	40
Control vs. lin-23(*ot1*)	1394	886.9	507.6	145.8	9	9	3.482	40
Control vs. *skn-1* RNAi	1394	823	571.4	145.8	9	9	3.92	40

bioRxiv preprint doi: https://doi.org/10.64898/2026.07.22.740106; this version posted July 26, 2026. The copyright holder for this preprint (which was not certified by peer review) is the author/funder, who has granted bioRxiv a license to display the preprint in perpetuity. It is made available under a CC-BY 4.0 International license.

Figure 1E

gst-4 Representative Values

Δ Ct values

WT (*E. coli)*	WT	*lin-23(ot1)*	*nipi-3(fr4)*
8.83	5.126667	5.99962636	7.00094126
8.75	4.837999	7.04609915	6.99666667
8.32	4.566667	6.51	5.98666667

gst-4 Representative Graph

Fold change

WT (*E. coli)*	WT	*lin-23(ot1)*	*nipi-3(fr4)*
1	12.99	7.09436121	3.54394912
1	15.00846	3.24811756	3.18950675
1	13.45434	3.49833068	5.0280535

PRISM ANALYSIS

Number of families	1
Number of comparisons per family	2
Alpha	0.05

Dunnett's multiple comparisons test	Mean diff.	95.00% CI of diff.	Below threshold?	Summary	Adjusted P Value	B-?	
WT vs. *lin-23(ot1)*	-1.675	-2.799 to -0.5507	Yes	**	0.0094	C	*lin-23(ot1)*
WT vs. *nipi-3(fr4)*	-1.818	-2.942 to -0.6935	Yes	**	0.0064	D	*nipi-3(fr4)*

Test details	Mean 1	Mean 2	Mean diff.	SE of diff.	n1	n2	q	DF
WT vs. *lin-23(ot1)*	4.844	6.519	-1.675	0.3926	3	3	4.266	6
WT vs. *nipi-3(fr4)*	4.844	6.661	-1.818	0.3926	3	3	4.629	6

bioRxiv preprint doi: https://doi.org/10.64898/2026.07.22.740106; this version posted July 26, 2026. The copyright holder for this preprint (which was not certified by peer review) is the author/funder, who has granted bioRxiv a license to display the preprint in perpetuity. It is made available under a CC-BY 4.0 International license.

Figure 1F

gcs-1 Representative Values

Δ Ct values

WT (*E. coli*)	WT	*lin-23(ot1)*	*nipi-3(fr4)*
6.68788816	5.743972	6.4450875	5.99692717
7.19120865	5.38847	6.70158614	6.04
7.47	5.496667	6.63333333	6.525

gcs-1 Representative Graph

Fold change

WT (*E. coli*)	WT	*lin-23(ot1)*	*nipi-3(fr4)*
1	1.923743	1.18328751	1.6143585
1	3.488818	1.40407744	1.2397077
1	3.926743	0.82169031	1.92518889

PRISM ANALYSIS

Number of families	1
Number of comparisons per family	2
Alpha	0.05

Dunnett's multiple comparisons test	Mean diff.	95.00% CI of diff.	Below threshold?	Summary	Adjusted P Value	B-?	
WT vs. *lin-23(ot1)*	-1.05	-1.550 to -0.5510	Yes	**	0.0017	C	*lin-23(ot1)*
WT vs. *nipi-3(fr4)*	-0.6443	-1.144 to -0.1450	Yes	*	0.018	D	*nipi-3(fr4)*

Test details	Mean 1	Mean 2	Mean diff.	SE of diff.	n1	n2	q	DF
WT vs. *lin-23(ot1)*	5.543	6.593	-1.05	0.1744	3	3	6.023	6
WT vs. *nipi-3(fr4)*	5.543	6.187	-0.6443	0.1744	3	3	3.695	6

bioRxiv preprint doi: https://doi.org/10.64898/2026.07.22.740106; this version posted July 26, 2026. The copyright holder for this preprint (which was not certified by peer review) is the author/funder, who has granted bioRxiv a license to display the preprint in perpetuity. It is made available under a CC-BY 4.0 International license.

Figure 2A

Trial #	Treatment	Median survival (days)	Statistics
Data for Figure 2a: Loss of LIN-23 decreases *C. elegans* survival			
1	wt	7	
2	wt	8	
3	wt	6	
			p-value* vs. *control
1	*lin23(ot1)*	5	$p < 0.0001$
2	*lin23(ot1)*	6	$p < 0.0001$
3	*lin23(ot1)*	5	$p = 0.0003$
1	Control RNAi	6	
2	Control RNAi	7	
3	Control RNAi	7	
			p-value* vs. *control
1	*lin23* RNAi	7	$p = 0.0104$
2	*lin23* RNAi	6	$p = 0.0218$
3	*lin23* RNAi	6	$p = 0.0419$

bioRxiv preprint doi: https://doi.org/10.64898/2026.07.22.740106; this version posted July 26, 2026. The copyright holder for this preprint (which was not certified by peer review) is the author/funder, who has granted bioRxiv a license to display the preprint in perpetuity. It is made available under a CC-BY 4.0 International license.

Figure 2B

Trial #	Treatment	Median survival (days)	Statistics	
**Data for Figure 2b: Loss of LIN-23 decreases *C. elegans* survival**				
1	*wt*	6		
2	*wt*	7		
3	*wt*	10		
			p-value* vs. *control	
1	*lin23* RNAi	4	p = 0.0002	
2	*lin23* RNAi	5	p < 0.0001	
3	*lin23* RNAi	9	p = 0.0006	
			p-value* vs. *control	
1	*skn1(zu135)*	5	p < 0.0001	
2	*skn1(zu135)*	5	p < 0.0001	
3	*skn1(zu135)*	7	p < 0.0001	
			p-value* vs. *control	***p-value* vs. *skn1(zu135)***
1	*skn1(zu135); lin23 RNAi*	5	p = 0.0023	p = 0.1165
2	*skn1(zu135); lin23 RNAi*	5	p < 0.0001	p = 0.0911
3	*skn1(zu135); lin23 RNAi*	6	p < 0.0001	p = 0.2488

bioRxiv preprint doi: https://doi.org/10.64898/2026.07.22.740106; this version posted July 26, 2026. The copyright holder for this preprint (which was not certified by peer review) is the author/funder, who has granted bioRxiv a license to display the preprint in perpetuity. It is made available under a CC-BY 4.0 International license.

Figure 3A

**Trial #**	**Treatment**	**Median survival (days)**	**Statistics**
Data for Figure 3A :			
1	*N2; control RNAi*	6	
2	*N2; control RNAi*	6	
3	*N2; control RNAi*	4	
			p-value* vs. *control
1	*N2;lin23 RNAi*	5	p < 0.0001
2	*N2;lin23 RNAi*	5	p = 0.0099
3	*N2;lin23 RNAi*	4	p = 0.0009
			p-value* vs. *control
1	*lin23(ot1)*	6	p < 0.0001
2	*lin23(ot1)*	5	p = 0.0005
3	*lin23(ot1)*	3.5	p < 0.0001
			p-value* vs. *control
1	*skn1(zu135)*	3	p < 0.0001
2	*skn1(zu135)*	2	p < 0.0001
3	*skn1(zu135)*	2	p < 0.0001
			p-value* vs. *control
1	*skn1(zu135);lin23 RNAi*	3	p < 0.0001
2	*skn1(zu135);lin23 RNAi*	2	p < 0.0001
3	*skn1(zu135);lin23 RNAi*	2	p < 0.0001

bioRxiv preprint doi: https://doi.org/10.64898/2026.07.22.740106; this version posted July 26, 2026. The copyright holder for this preprint (which was not certified by peer review) is the author/funder, who has granted bioRxiv a license to display the preprint in perpetuity. It is made available under a CC-BY 4.0 International license.

Figure 3B

gst-4 Representative Values

Δ Ct values

WT (*E. coli)*	WT	WT; *lin-23* RNAi	*lin-23(ot1)*	*skn-1(zu135)*	*skn-1(zu135); lin-23* RNAi
5.88	0.28	1.37333333	1.53333333	1.57	1.78333333
7.09333333	0.716667	1.37	0.83	2.16333333	1.75333333
7	0.91	1.17333333	1.17333333	1.55666667	2.32666667
6.74666667	0.776667	1.14666667	0.93	1.75333333	1.81666667

gst-4 Representative Graph

Fold change

WT (*E. coli)*	WT	WT; *lin-23* RNAi	*lin-23(ot1)*	*skn-1(zu135)*	*skn-1(zu135); lin-23* RNAi
1	48.50293	22.7322198	20.3459067	19.8353232	17.1088
1	83.09367	52.8317515	76.8159147	30.4844159	40.504211
1	68.11969	56.7546489	56.7546489	43.5117559	25.516054
1	62.6829	48.5029301	56.3626159	31.8524697	30.4844159

PRISM ANALYSIS

Number of families	1
Number of comparisons per family	5
Alpha	0.05

Dunnett's multiple comparisons test	Mean diff.	95.00% CI of diff.	Below threshold?	Summary	Adjusted P Value	B-?	
N2 vs. N2 *E. coli*	-6.009	-6.651 to -5.368	Yes	****	<0.0001	A	N2 *E. coli*
N2 vs. N2 *in-23* RNAi	-0.595	-1.236 to 0.04649	No	ns	0.0743	C	N2 *in-23* RNAi
N2 vs. *lin-23(ot1)*	-0.4458	-1.087 to 0.1957	No	ns	0.2375	D	*lin-23(ot1)*
N2 vs. *skn-1(zu135)*	-1.09	-1.731 to -0.4485	Yes	***	0.0008	E	*skn-1(zu135)*
N2 vs. *skn-1(zu135) lin-23* RNAi	-1.249	-1.891 to -0.6077	Yes	***	0.0002	F	*skn-1(zu135) lin-23* RNAi

Test details	Mean 1	Mean 2	Mean diff.	SE of diff.	n1	n2	q	DF
N2 vs. N2 *E. coli*	0.6708	6.68	-6.009	0.2323	4	4	25.87	18
N2 vs. N2 *in-23* RNAi	0.6708	1.266	-0.595	0.2323	4	4	2.561	18
N2 vs. *lin-23(ot1)*	0.6708	1.117	-0.4458	0.2323	4	4	1.919	18
N2 vs. *skn-1(zu135)*	0.6708	1.761	-1.09	0.2323	4	4	4.692	18
N2 vs. *skn-1(zu135) lin-23* RNAi	0.6708	1.92	-1.249	0.2323	4	4	5.377	18

bioRxiv preprint doi: https://doi.org/10.64898/2026.07.22.740106; this version posted July 26, 2026. The copyright holder for this preprint (which was not certified by peer review) is the author/funder, who has granted bioRxiv a license to display the preprint in perpetuity. It is made available under a CC-BY 4.0 International license.

Figure 3C

gcs-1 Representative Values

Δ Ct values

WT (*E. coli)*	WT	WT; *lin-23* RNAi	*lin-23(ot1)*	*skn-1(zu135)*	*skn-1(zu135); lin-23* RNAi
6.07	1.436667	2.67666667	2.76666667	3.25	3.19333333
6.97333333	2.2	2.53	2.32333333	3.49	2.87
7.09666667	2.296667	2.51	2.71	3.13666667	3.64333333
7.02333333	2.19	2.50333333	2.65	3.50333333	3.29333333

gcs-1 Representative Graph

Fold change

WT (*E. coli)*	WT	WT; *lin-23* RNAi	*lin-23(ot1)*	*skn-1(zu135)*	*skn-1(zu135); lin-23* RNAi
1	24.81832	10.5073964	9.871938	7.06162397	7.34451216
1	27.34743	21.755878	25.1066911	11.1837595	17.1880424
1	27.85762	24.0283665	20.917908	15.5624792	10.953601
1	28.50876	22.943284	20.725476	11.471642	13.2691127

PRISM ANALYSIS

Number of families	1
Number of comparisons per family	5
Alpha	0.05

Dunnett's multiple comparisons test	Mean diff.	95.00% CI of diff.	Below threshold?	Summary	Adjusted P Value	B-?	
N2 vs. N2 *E. coli*	-4.76	-5.363 to -4.157	Yes	****	<0.0001	A	N2 *E. coli*
N2 vs. N2 *in-23* RNAi	-0.5242	-1.128 to 0.07932	No	ns	0.1015	C	N2 *in-23* RNAi
N2 vs. *lin-23(ot1)*	-0.5817	-1.185 to 0.02182	No	ns	0.061	D	*lin-23(ot1)*
N2 vs. *skn-1(zu135)*	-1.314	-1.918 to -0.7107	Yes	****	<0.0001	E	*skn-1(zu135)*
N2 vs. *skn-1(zu135) lin-23* RNAi	-1.219	-1.823 to -0.6157	Yes	***	0.0001	F	*skn-1(zu135) lin-23* RNAi

Test details	Mean 1	Mean 2	Mean diff.	SE of diff.	n1	n2	q	DF
N2 vs. N2 *E. coli*	2.031	6.791	-4.76	0.2185	4	4	21.78	18
N2 vs. N2 *in-23* RNAi	2.031	2.555	-0.5242	0.2185	4	4	2.399	18
N2 vs. *lin-23(ot1)*	2.031	2.613	-0.5817	0.2185	4	4	2.662	18
N2 vs. *skn-1(zu135)*	2.031	3.345	-1.314	0.2185	4	4	6.013	18
N2 vs. *skn-1(zu135) lin-23* RNAi	2.031	3.25	-1.219	0.2185	4	4	5.579	18

bioRxiv preprint doi: https://doi.org/10.64898/2026.07.22.740106; this version posted July 26, 2026. The copyright holder for this preprint (which was not certified by peer review) is the author/funder, who has granted bioRxiv a license to display the preprint in perpetuity. It is made available under a CC-BY 4.0 International license.

Figure 4A

Uncropped representative blot (WB #5)

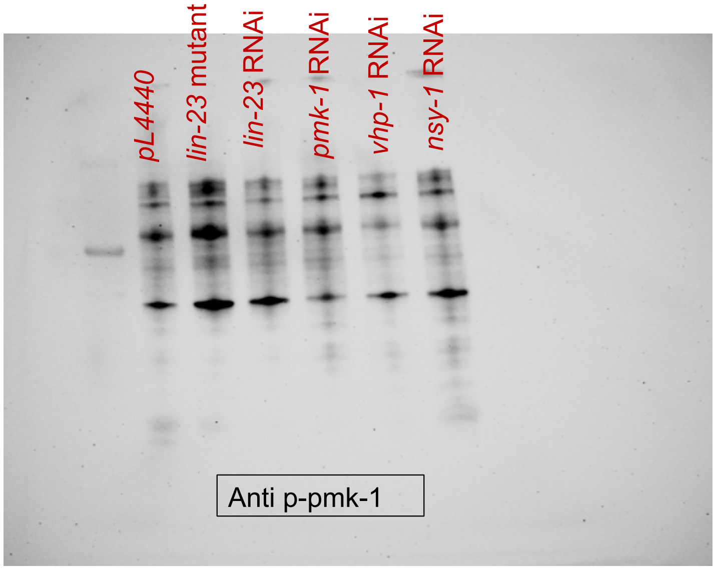

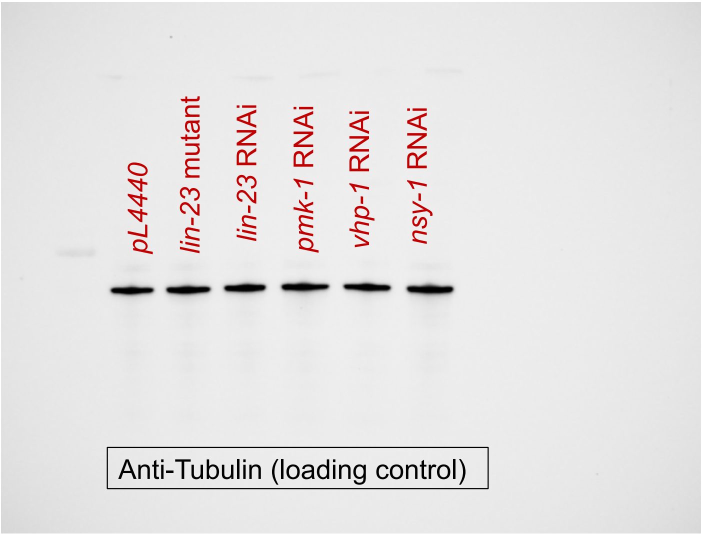

bioRxiv preprint doi: https://doi.org/10.64898/2026.07.22.740106; this version posted July 26, 2026. The copyright holder for this preprint (which was not certified by peer review) is the author/funder, who has granted bioRxiv a license to display the preprint in perpetuity. It is made available under a CC-BY 4.0 International license.

Figure 4B

Western blot values

Western Blot No.5

Sample	Fold change
Control	1
lin-23(ot1)	2.031637363
lin-23 RNAi	1.645654644
pmk-1 RNAi	0.785926462

Western Blot No.7

Sample	Fold change
Control	1
lin-23(ot1)	1.9284797
lin-23 RNAi	1.17398088
pmk-1 RNAi	0.82351699

Western Blot No.11

Sample	Fold change
Control	1
lin-23(ot1)	1.278565403
lin-23 RNAi	1.275154643
pmk-1 RNAi	0.558075902

PRISM ANALYSIS

Table Analyzed	WB E. faecalis results	
Group B	*lin-23(ot1)*	
vs.	vs.	
Group A	control	
Unpaired t test		
P value		0.034
P value summary	*	
Significantly different (P < 0.05)?	Yes	
One- or two-tailed P value?	Two-tailed	
t, df	t=3.166, df=4	

Table Analyzed	WB E. faecalis results	
Group C	*lin-23 RNAi*	
vs.	vs.	
Group A	control	
Unpaired t test		
P value		0.0636
P value summary	ns	
Significantly different (P < 0.05)?	No	
One- or two-tailed P value?	Two-tailed	
t, df	t=2.545, df=4	

Table Analyzed	WB E. faecalis results	
Group D	*pmk-1 RNAi*	
vs.	vs.	
Group A	control	
Unpaired t test		
P value		0.0287
P value summary	*	
Significantly different (P < 0.05)?	Yes	
One- or two-tailed P value?	Two-tailed	
t, df	t=3.346, df=4	

bioRxiv preprint doi: https://doi.org/10.64898/2026.07.22.740106; this version posted July 26, 2026. The copyright holder for this preprint (which was not certified by peer review) is the author/funder, who has granted bioRxiv a license to display the preprint in perpetuity. It is made available under a CC-BY 4.0 International license.

Figure 4D

SKN-1B/C::GFP Categorical Scoring	low	medium	high
Control n=54	10	23	21
lin-23(ot1) n=52	7	13	32
lin-23 RNAi n=55	1	17	37
skn-1 RNAi n=57	57	0	0

PRISM ANALYSIS

For OG1RF Exposure

Table Analyzed	lin-23(ot1) OG1RF
P value and statistical significance	
Test	Fisher's exact test
P value	0.0666
P value summary	ns
One- or two-sided	NA
Statistically significant (P < 0.05)?	No
Data analyzed	
Number of rows	2
Number of columns	3

Table Analyzed	lin-23 RNAi OG1RF
P value and statistical significance	
Test	Fisher's exact test
P value	0.0015
P value summary	**
One- or two-sided	NA
Statistically significant (P < 0.05)?	Yes
Data analyzed	
Number of rows	2
Number of columns	3

Table Analyzed	skn-1 RNAi OG1RF
P value and statistical significance	
Test	Fisher's exact test
P value	<0.0001
P value summary	****
One- or two-sided	NA
Statistically significant (P < 0.05)?	Yes
Data analyzed	
Number of rows	2
Number of columns	3

bioRxiv preprint doi: https://doi.org/10.64898/2026.07.22.740106; this version posted July 26, 2026. The copyright holder for this preprint (which was not certified by peer review) is the author/funder, who has granted bioRxiv a license to display the preprint in perpetuity. It is made available under a CC-BY 4.0 International license.

Figure 5B

Object mean GFP

gst-4 ::GFP representative graph values

RNAi Treatment	wdr-23 (tm1817) OP50		
Control	46921.3333	50691	56881
skn-1 RNAi	10979	17240.3333	22968
lin-23 RNAi	51627	41211.5	56888

PRISM ANALYSIS

E. coli Analysis

Compare each cell mean with the control (upper-left) cell mean

Number of families	1
Number of comparisons per family	5
Alpha	0.05

Dunnett's multiple comparisons test	Mean diff.	95.00% CI of diff.	Below threshold?	Summary	Adjusted P Value
Control :wdr-23 (tm1817) OP50 vs. *skn-1* :wdr-23 (tm1817) OP50	34435	18463 to 50408	Yes	***	0.0002
Control :wdr-23 (tm1817) OP50 vs. *lin-23* :wdr-23 (tm1817) OP50	1589	-14384 to 17562	No	ns	0.9983

Test details	Mean 1	Mean 2	Mean diff.	SE of diff.	N1	N2	q	DF
Control :wdr-23 (tm1817) OP50 vs. *skn-1* :wdr-23 (tm1817) OP50	51498	17062	34435	5505	3	3	6.255	12
Control :wdr-23 (tm1817) OP50 vs. *lin-23* :wdr-23 (tm1817) OP50	51498	49909	1589	5505	3	3	0.2886	12

bioRxiv preprint doi: https://doi.org/10.64898/2026.07.22.740106; this version posted July 26, 2026. The copyright holder for this preprint (which was not certified by peer review) is the author/funder, who has granted bioRxiv a license to display the preprint in perpetuity. It is made available under a CC-BY 4.0 International license.

Figure 5C

Object mean GFP

gst-4 ::GFP representative graph values

RNAi Treatment	wdr-23 (tm1817) OG1RF		
Control	57186.5	38860	50256.33
skn-1 RNAi	28449.3333	25396	23436
lin-23 RNAi	54227	46375	39284.5

PRISM ANALYSIS

***E. faecalis* Analysis**

Compare each cell mean with the control (upper-left) cell mean

Number of families	1
Number of comparisons per family	5
Alpha	0.05

Dunnett's multiple comparisons test	Mean diff.	95.00% CI of diff.	Below threshold?	Summary	Adjusted P Value
Control :wdr-23 (tm1817) OG1RF vs. *skn-1* :wdr-23 (tm1817) OG1RF	23007	7035 to 38980	Yes	**	0.0052
Control :wdr-23 (tm1817) OG1RF vs. *lin-23* :wdr-23 (tm1817) OG1RF	2139	-13834 to 18111	No	ns	0.9934

Test details	Mean 1	Mean 2	Mean diff.	SE of diff.	N1	N2	q	DF
Control :wdr-23 (tm1817) OG1RF vs. *skn-1* :wdr-23 (tm1817) OG1RF	48768	25760	23007	5505	3	3	4.179	12
Control :wdr-23 (tm1817) OG1RF vs. *lin-23* :wdr-23 (tm1817) OG1RF	48768	46629	2139	5505	3	3	0.3885	12

bioRxiv preprint doi: https://doi.org/10.64898/2026.07.22.740106; this version posted July 26, 2026. The copyright holder for this preprint (which was not certified by peer review) is the author/funder, who has granted bioRxiv a license to display the preprint in perpetuity. It is made available under a CC-BY 4.0 International license.

Figure 5D

gst-4 Representative Values

Δ Ct values

WT *E. coli*	WT	lin-23(ot1); wdr-23(tm1817)	lin-23(ot1)	wdr-23(tm1817)
7.60673297	2.96437769	-1.8840117	4.12954571	-1.7544706
5.45666667	3.60666667	-1.12	4.61	-1.7466667
5.04666667	1.06666667	-2.4366667	2.33	-2.2433333

gst-4 Representative Graph

Fold change

WT *E. coli*	WT	lin-23(ot1); wdr-23(tm1817)	lin-23(ot1)	wdr-23(tm1817)
1	24.97400468	719.4470329	11.13621646	657.6625032
1	3.60500185	95.44956163	1.798341071	147.3735015
1	15.77972327	178.9401516	6.573522517	156.4979555

PRISM ANALYSIS

Number of families	1
Number of comparisons per family	2
Alpha	0.05

Dunnett's multiple comparisons test	Mean diff.	95.00% CI of diff.	Below threshold?	Summary	Adjusted P Value	B-?	
lin-23(ot1); wdr-23(tm1817 vs. lin-23(ot1)	-5.503	-7.394 to -3.613	Yes	***	0.0003	C	lin-23(ot1)
lin-23(ot1); wdr-23(tm1817 vs. wdr-23(tm1817)	0.1013	-1.790 to 1.992	No	ns	0.983	D	wdr-23(tm1817)

Test details	Mean 1	Mean 2	Mean diff.	SE of diff.	n1	n2	q	DF
lin-23(ot1); wdr-23(tm1817 vs. lin-23(ot1)	-1.814	3.69	-5.503	0.6604	3	3	8.333	6
lin-23(ot1); wdr-23(tm1817 vs. wdr-23(tm1817)	-1.814	-1.915	0.1013	0.6604	3	3	0.1533	6

bioRxiv preprint doi: https://doi.org/10.64898/2026.07.22.740106; this version posted July 26, 2026. The copyright holder for this preprint (which was not certified by peer review) is the author/funder, who has granted bioRxiv a license to display the preprint in perpetuity. It is made available under a CC-BY 4.0 International license.

Figure 5E

gcs-1 Representative Values

Δ Ct values

WT *E. coli*	WT	lin-23(ot1); wdr-23(tm1817)	lin-23(ot1)	wdr-23(tm1817)
6.14006861	4.97994862	1.77715478	5.55426677	1.99683965
4.76333333	4.36666667	-0.2433333	4.54666667	-0.0333333
5.40333333	2.70333333	0.21333333	4.82333333	0.07

gcs-1 Representative Graph

Fold change

WT *E. coli*	WT	lin-23(ot1); wdr-23(tm1817)	lin-23(ot1)	wdr-23(tm1817)
1	2.234760135	20.5763307	1.500872939	17.66998569
1	1.316462719	32.14821358	1.162045587	27.79332756
1	6.498019171	36.50443891	1.494849249	40.3174736

PRISM ANALYSIS

Number of families	1
Number of comparisons per family	2
Alpha	0.05

Dunnett's multiple comparisons test	Mean diff.	95.00% CI of diff.	Below threshold?	Summary	Adjusted P Value	B-?	
lin-23(ot1); wdr-23(tm1817 vs. lin-23(ot1)	-4.392	-6.611 to -2.174	Yes	**	0.0023	C	lin-23(ot1)
lin-23(ot1); wdr-23(tm1817 vs. wdr-23(tm1817)	-0.09545	-2.314 to 2.123	No	ns	0.9889	D	wdr-23(tm1817)

Test details	Mean 1	Mean 2	Mean diff.	SE of diff.	n1	n2	q	DF
lin-23(ot1); wdr-23(tm1817 vs. lin-23(ot1)	0.5824	4.975	-4.392	0.7748	3	3	5.669	6
lin-23(ot1); wdr-23(tm1817 vs. wdr-23(tm1817)	0.5824	0.6778	-0.09545	0.7748	3	3	0.1232	6

bioRxiv preprint doi: https://doi.org/10.64898/2026.07.22.740106; this version posted July 26, 2026. The copyright holder for this preprint (which was not certified by peer review) is the author/funder, who has granted bioRxiv a license to display the preprint in perpetuity. It is made available under a CC-BY 4.0 International license.

Figure S1B

SKN-1B/C::GFP Categorical Scoring	low	medium	high
Control<br>n=98	9	38	51
lin-23(ot1)<br>n=95	6	47	52
lin-23 *RNAi*<br>n=103	10	38	55
skn-1 *RNAi*<br>n=91	91	0	0

PRISM ANALYSIS

For PA14 Exposure

Table Analyzed	lin-23(ot1) PA14
P value and statistical significance	
Test	Fisher's exact test
P value	0.5529
P value summary	ns
One- or two-sided	NA
Statistically significant (P < 0.05)?	No
Data analyzed	
Number of rows	2
Number of columns	3

Table Analyzed	lin-23 RNAi PA14
P value and statistical significance	
Test	Fisher's exact test
P value	0.9775
P value summary	ns
One- or two-sided	NA
Statistically significant (P < 0.05)?	No
Data analyzed	
Number of rows	2
Number of columns	3

Table Analyzed	skn-1 RNAi PA14
P value and statistical significance	
Test	Fisher's exact test
P value	<0.0001
P value summary	****
One- or two-sided	NA
Statistically significant (P < 0.05)?	Yes
Data analyzed	
Number of rows	2
Number of columns	3
